# Peptides reloaded - new strategies for tolerogenic vaccination

**DOI:** 10.1186/ar3407

**Published:** 2011-09-16

**Authors:** Alf Hamann, Jennifer Pfeil, Elisabeth Kenngott, Tony Pernthaner, Susanne Hartmann, Ute Hoffmann

**Affiliations:** 1 Experimental Rheumatology, Charite Universitätsmedizin and Deutsches Rheuma-Forschungszentrum Berlin, Germany; 2 AgResearch, Hopkirk Research Institute, Massey University, Palmerston North, New Zealand; 3 Inst. Parasitology, Humboldt University Berlin, Germany

## 

Uncontrolled immune reactions, e.g. in autoimmunity, chronic inflammation or allergy are a major cause of chronic and partially life-threatening diseases. Current treatments including those involving biologics largely rely on unspecific suppression of the effector cells and rarely are able to cure the disease. The native mechanisms of tolerance, notably those of active suppression by regulatory cells, have therefore fascinated immunologists from the beginning on as they promise modulation of the immune system in an antigen-specific way. However, early attempts to achieve tolerance by oral immunization or peptide vaccination worked in mouse models, but hardly were successful in humans. This might have two reasons: a), the modes of tolerogenic vaccination might not be very efficient, and b), the abundance of inflammatory effector/memory cells in adult humans might prevent induction and functioning of regulatory cells.

Our group is presently focusing on the first point and designing novel modifications of peptide-based vaccines able to induce regulatory cells. Conjugation of peptides to carrier molecules is one way to improve their in vivo efficacy in inducing Foxp3+ Tregs. In an EAE model, an improved protective efficacy can be demonstrated. A second approach is aiming to target the antigen to the gastrointestinal route which is usually associated with tolerization rather than effector response. Use of signal molecules targeting the peptides to epithelial transport mechanisms is presently explored to improve the efficacy of intestinal vaccination. Finally, we investigate immunomodulatory substances produced by parasites for a potential use as tolerogenic adjuvants. While these approaches might help to improve feasibility of peptide vaccination to induce tolerance, we assume that treatment of existing autoimmune disease will require a combination therapy which incorporates elimination of inflammatory effector cells or suppression of their activity.

